# Design and Characterization of Maltoheptaose-*b*-Polystyrene Nanoparticles, as a Potential New Nanocarrier for Oral Delivery of Tamoxifen

**DOI:** 10.3390/molecules26216507

**Published:** 2021-10-28

**Authors:** Marcos Antonio Villetti, Adryana Rocha Clementino, Ilaria Dotti, Patricia Regina Ebani, Eride Quarta, Francesca Buttini, Fabio Sonvico, Annalisa Bianchera, Redouane Borsali

**Affiliations:** 1 Laboratório de Espectroscopia e Polímeros (Lepol), Departamento de Física, Universidade Federal de Santa Maria, Santa Maria 97105-900, Brazil; mvilletti@ufsm.br (M.A.V.); patricia.ebani@ufsm.br (P.R.E.); 2 Biopharmanet-TEC, University of Parma, 43124 Parma, Italy; adryana.rochaclementino@studenti.unipr.it (A.R.C.); francesca.buttini@unipr.it (F.B.); fabio.sonvico@unipr.it (F.S.); 3 Department of Food and Drug, University of Parma, 43124 Parma, Italy; ilaria.dotti@studenti.unipr.it (I.D.); eride.quarta@studenti.unipr.it (E.Q.); 4 Department of Chemistry, University Grenoble Alpes, CNRS, CERMAV, 38000 Grenoble, France

**Keywords:** tamoxifen citrate, block copolymer, cytotoxicity, breast cancer

## Abstract

Tamoxifen citrate (TMC), a non-steroidal antiestrogen drug used for the treatment of breast cancer, was loaded in a block copolymer of maltoheptaose-*b*-polystyrene (MH-*b*-PS) nanoparticles, a potential drug delivery system to optimize oral chemotherapy. The nanoparticles were obtained from self-assembly of MH-b-PS using the standard and reverse nanoprecipitation methods. The MH-b-PS@TMC nanoparticles were characterized by their physicochemical properties, morphology, drug loading and encapsulation efficiency, and release kinetic profile in simulated intestinal fluid (pH 7.4). Finally, their cytotoxicity towards the human breast carcinoma MCF-7 cell line was assessed. The standard nanoprecipitation method proved to be more efficient than reverse nanoprecipitation to produce nanoparticles with small size and narrow particle size distribution. Moreover, tamoxifen-loaded nanoparticles displayed spherical morphology, a positive zeta potential and high drug content (238.6 ± 6.8 µg mL^−1^) and encapsulation efficiency (80.9 ± 0.4 %). In vitro drug release kinetics showed a burst release at early time points, followed by a sustained release profile controlled by diffusion. MH-b-PS@TMC nanoparticles showed higher cytotoxicity towards MCF-7 cells than free tamoxifen citrate, confirming their effectiveness as a delivery system for administration of lipophilic anticancer drugs.

## 1. Introduction

**Figure 1:**
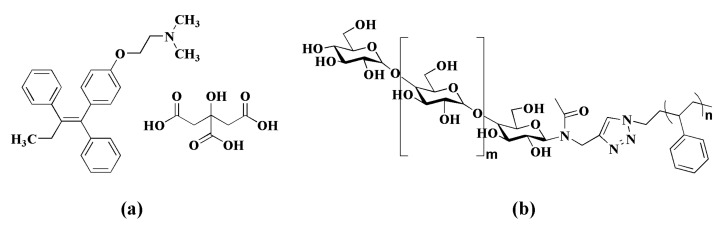
Chemical structures of (**a**) tamoxifen citrate and (**b**) maltoheptaose-*b*-polystyrene (MH-b-PS).

Recently, in the field of oncology, special attention has been paid to the potential use of alternative routes to parenteral administration of anticancer drugs in order to improve the quality of life of patients. In this sense, the oral route has gained prominence due to its convenience, reduced side-effects, and also because generally it does not require hospitalization of the patient [1]. Tamoxifen citrate (TMC) (Figure 1a) is a non-steroidal antiestrogen drug, administered orally, and primarily indicated for the treatment and prevention of estrogen-dependent breast cancer in pre- and post-menopausal women. Tamoxifen has a very complex metabolism in the human organism, involving many active metabolites, which exhibit more antiestrogenic effects in breast cancer cells than their precursor [2]. Briefly, in the first step, tamoxifen is metabolized by cytochrome P-450 enzymes into 4-hydroxy-tamoxifen (7%), N-desmethyl-tamoxifen (92%), and other metabolites [3,4,5,6]. Then, in a second step, these metabolites are converted into endoxifen (N-desmethyl-4-hydroxytamoxifen), which is around 100-fold more potent as an antagonist of estrogen receptor than tamoxifen [6]. Endoxifen’s mechanism of action is dual—as an aromatase inhibitor and as a selective estrogen receptor modulator—thereby inhibiting the growth of malignant breast cells.

Although tamoxifen has a well-established anti-tumoral activity, its efficacy is somewhat limited by important intra- and inter-patient variability in oral bioavailability [7]. This clinical evidence is explained by the drug’s low water solubility and susceptibility to enzymatic degradation [8], which decrease its gastrointestinal absorption. In this sense, nanoscale vehicles (polymeric nanoparticles, micelles, liposomes, etc.) have been proposed to encapsulate tamoxifen in order to improve its biopharmaceutical properties, such as apparent aqueous solubility, permeability across intestinal mucosa and cell uptake, and to reduce its systemic adverse effects [9,10,11,12,13]. The major challenge is to develop novel nanocarriers able to tune the intestinal absorption of TMC, aiming to improve its therapeutic efficacy in oral administration. Recently, nanoparticles based on maltoheptaose copolymers have emerged as a potential nanocarrier for hydrophobic guest molecules such as progesterone [14] and Nile red dye [15], to encapsulate gold nanoparticles [16], and stand out for their application in different branches of nanotechnology [17,18,19,20].

Maltoheptaose-based copolymers belong to an attractive class of temperature-responsive polymers that self-assemble in a specific solvent to create nanostructured materials with morphological characteristics intrinsically linked to their architecture and composition [21,22,23]. Self-assembly of maltoheptaose-based copolymers occur because of their amphiphilic nature, deriving from the combination of hydrophilic blocks of maltoheptaose with hydrophobic blocks of polystyrene (PS) [24], polymethylmethacrylate (PMMA) [25], and polycaprolactone (PCL) [14]. Then, when a maltoheptaose-based copolymer is dissolved in a solvent that is thermodynamically good for one block but poor for the other, spontaneous self-assembling occurs, leading to the formation of a core-shell structure with the solvophobic block forming the core and the solvophilic part forming the shell of the nanoparticle. Another important feature of the maltoheptaose-based copolymer is that it has suitable characteristics to interact with different target cancer cells: actually, many cancer cells are known to have increased metabolism and overexpression of glucose transporters, a phenomenon known as Warburg effect [26]. The presence of glucose units in the structure of the copolymer could constitute a potential targeting strategy for the delivery of chemotherapeutic drugs to breast cancer cells [27] as well as to other kinds of tumors. Therefore, the study of new nanostructured systems is necessary due to the lack of information regarding the physicochemical and biopharmaceutical properties of the TMC-loaded maltoheptaose-based copolymer.

Polystyrene was used as a hydrophobic block in the copolymer since it is safe and biocompatible because it does not degrade in the cellular environment and exhibits no short-term cytotoxicity [28]. Moreover, the use of polystyrene in the core of nanoparticles such as in polyethylene glycol (PEG) tethered mannose (M)-block-PS (PEG-M-b-PS) did not exhibit consistent significant levels of toxicity towards human lung and dermal fibroblasts and rat lung macrophages, evidencing that these nanoparticles may be safe for use as carriers for antibacterial agents [29]. To date, there are no reports in the literature on the use of amphiphilic block copolymer consisting of maltoheptaose-*b*-polystyrene (Figure 1b) for the encapsulation of the antiestrogen drug tamoxifen citrate. The goal was to develop tamoxifen-loaded maltoheptaose-*b*-polystyrene nanoparticles (MH-b-PS@TMC) aiming to surpass the drawbacks associated with poor TMC solubility in biological medium and control the rate of drug release. The present work describes MH-b-PS@TMC manufacturing by the standard method (organic phase is added to the aqueous phase) and reverse nanoprecipitation methods (aqueous phase is added to the organic phase). Several manufacturing parameters were evaluated to optimize the physico–chemical and biopharmaceutical properties of the nanoparticles, such as content of oil, drug, copolymer and mixture of solvents (THF/H_2_O), temperature, filtration, and use of ultrasound. This is the first report on the development of MH-b-PS@TMC nanoparticles with a detailed investigation of its physicochemical properties, morphology, drug content and encapsulation efficiency, drug release profile in simulated intestinal fluid (pH 7.4), and cytotoxicity towards the human breast carcinoma cell line MCF-7.

## 2. Results and Discussion

### 2.1. Formulations and Physicochemical Properties of MH-b-PS@TMC Nanoparticles

The MH-*b*-PS block copolymer, due to its amphiphilic nature, can self-organize when placed in specific solvents (suitable to dissolve one block only), leading to the formation of polymeric micelles [24]. In this work, the THF/water (8:2 *w*/*w*) mixture was used to disperse the MH-b-PS block copolymer, since it is a good solvent for both MH and PS blocks and yields to swollen single-chains of MH-b-PS in solution with a diameter size of 5 nm [19]. Then, nanoparticles were obtained using reverse and standard nanoprecipitation method, in which 11 formulations were prepared and the best condition was evaluated to obtain thermodynamically stable dispersions. It is noteworthy that preliminary experiments evidenced that the nanoparticles containing both oil and TMC in the formulation were found to be stable, whereas nanoparticles prepared only with TMC drug were instable (data not shown). This outcome highlights the role of the oil in stabilizing the structure and hydrophobic interactions, leading to the encapsulation of the drug in the core of nanoparticles. In the manufacturing process, several parameters were evaluated in order to obtain stable formulations, such as the content of oil, drug, and copolymer, the ratio of solvents (THF/H_2_O), temperature, filtration, and use of ultrasound. A detailed protocol for preparation of MH-b-PS@TMC nanoparticles and the composition of formulations are available in Supplementary Material (see Tables S1 and S2).

Following the preparation, stable dispersions of nanoparticles were obtained showing a homogeneous appearance. The nanoparticles prepared by reverse nanoprecipitation method (NP-1 to NP-6) looked like a milky white, opalescent fluid, while those obtained by the standard method (NP-7 to NP-11) presented a bluish opalescent aspect (almost transparent). This outcome can be explained considering that the Tyndall effect depends on the size of the nanoparticles and suggests that the reverse nanoprecipitation method leads to the production of larger polymeric micelles when compared to the standard method. To confirm this hypothesis, the hydrodynamic behavior of polymeric micelles was analyzed by means of DLS; the results are shown in Table 1. As can be seen, nanoparticles obtained by reverse nanoprecipitation present a larger size and higher polydispersity index (PDI) with respect to those obtained by the standard method, explaining the different visual appearance of formulations and indicating that the preparation method plays an important role on their physicochemical properties. On the other hand, the ζ potential values were all positive and relatively high (see Table 1) for particles prepared with both methods, confirming the good thermodynamic stability of the colloidal dispersions. In addition, the difference in ζ potential values evidenced between NP-11 (+21.5 mV) and Blank NP-11 (−11.7 mV) indicates that the drug TMC may be loaded not only within the hydrophobic core of the nanoparticle but also on the surface, reversing the overall particle surface charge. This inversion of the ζ potential of the nanoparticles with the addition of TMC is in line with previous studies conducted on lecithin vesicles [10], PEG-PPG-PEG triblock micelles [9], poly(lactide-co-glycolide) (PLGA) [11], poly-ε-caprolactone (PCL) [11], and chitosan nanoparticles [11].

The physicochemical properties of nanoparticles are keys to treatment outcomes in oral drug delivery and can be tailored to bypass biological barriers to achieve therapeutic efficacy. The size and surface properties of nanoparticles are of outmost importance to pass through the intestinal mucus layer [30,31] and for their uptake by intestinal epithelial cells (enterocytes and M cells), and are a critical determinant of orally delivered nanoparticle fate [32,33]. It is generally accepted that the decrease in particle size increases the transcellular transport of nanoparticles by transcytosis. For PLGA nanoparticles 100 nm in size, the efficiency of uptake by intestinal tissue was 15–250 fold higher, compared to larger particles (500 nm, 1 μm, and 10 μm), and the permeability was dependent on the type and location of the tissue collected (duodenum or ileum) [34]. In the same way, in vitro investigation showed enhanced cellular uptake efficiency by Caco-2 cells for PS nanoparticles 100 nm in size, when compared to the other sizes (200 and 500 nm) [35]. Likewise, nanoparticles that exhibit small sizes (between 20 and 200 nm) are more suitable for accumulation in tumor target with respect to normal cells due to a phenomenon known as the enhanced permeability and retention (EPR) effect [36,37]. Bearing this in mind, the polymeric nanoparticles prepared by the standard method are expected to be more promising than those obtained by reverse nanoprecipitation for oral administration.

Among nanoparticles prepared by the standard nanoprecipitation method, NP-11 were chosen for carrying out further studies due to its small size, low PDI, and appropriate zeta potential (+21.5 mV) to ensure stability of colloidal dispersion. Thereby, a more in-depth investigation of the physicochemical properties of NP-11 (MH-b-PS@TMC) and Blank NP-11 (MH-b-PS) was performed using multi-angle light scattering, nanoparticle tracking analysis (NTA) and atomic force microscopy (AFM). Then, drug content and encapsulation efficiency, tamoxifen release kinetics, and cytotoxicity towards MCF-7 cells were evaluated as well.

**Figure 2:**
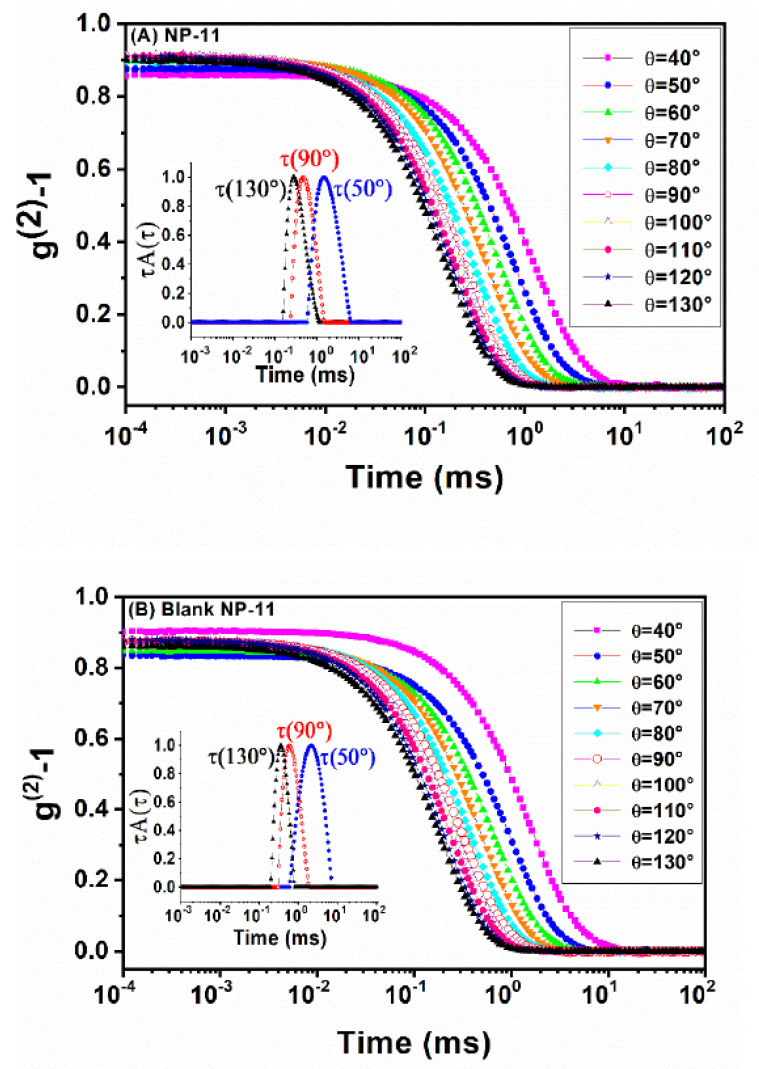
Intensity autocorrelation function (g^(2)^−1) measured at multiangle (θ) for (**A**) NP-11 and (**B**) blank NP-11. Inserts present the relaxation-time distribution of each sample at scattering angles of 50°, 90°, and 130°.

Multiangle light scattering measurements provide more robust, reproducible, and accurate data than single-angle analysis, particularly for multimodal samples, since they include autocorrelation functions for different scattering angles (θ) [38,39], and give a more accurate determination of the diffusion coefficient (D) [15]. Figure 2A,B shows the intensity autocorrelation function measured at multiangles (40°≤θ≤130°) for NP-11 and blank NP-11, respectively, and the inset shows the relaxation-time distribution at scattering angles of 50°, 90°, and 130°. As can be seen, the relaxation time distribution curves are monomodal and a relatively narrow size distribution is observed, confirming the existence of only one population. Similar behavior on size distribution was observed at another scattering angle (results not shown here). Moreover, no peaks were observed at higher relaxation times, at other scattering angles, indicating good homogeneity of the MH-b-PS nanoparticles. The autocorrelation functions were fitted by employing the CONTIN method and relaxation frequencies ($\Gamma \left(s^{-1}\right))$ were obtained for each scattering angle.

**Figure 3:**
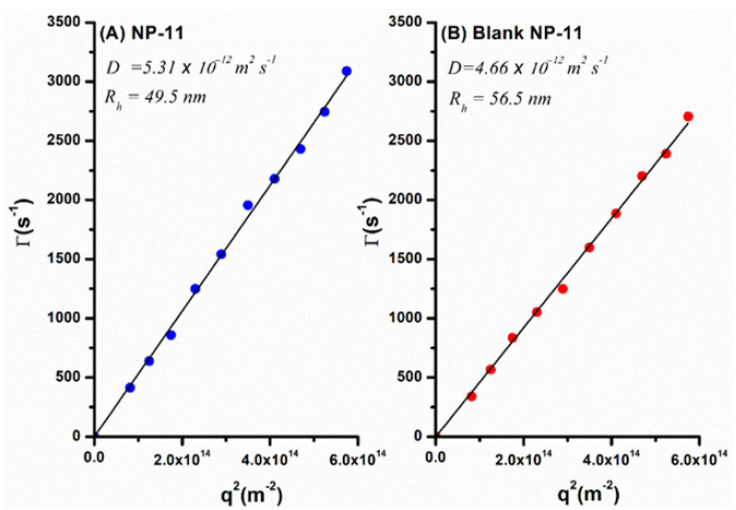
Dependence of relaxation frequency Γ ($s^{-1}$) on the square of wave vector modulus (q^2^) for (**A**) NP-11 and (**B**) blank NP-11.

As shown in Figure 3, the relaxation mode for NP-11 and blank NP-11 is diffusive since a linear relationship between $\Gamma \left(s^{-1}\right)$ and square of wave vector modulus ($q^{2}$) (passing through the origin) was observed, which can be attributed to translational diffusion of the nanoparticles in the medium. The mean hydrodynamic radii ($R_{h}$) of the nanoparticles were calculated from the diffusion coefficient ($\Gamma =Dq^{2}$), using the Stokes−Einstein relation. The resulting mean hydrodynamic diameters ($D_{h}=2R_{h}$) were 99 and 113 nm for NP-11 and blank NP-11, respectively, indicating that the nanoparticles containing TMC display a smaller size but faster diffusion than drug-free nanoparticles.

**Figure 4:**
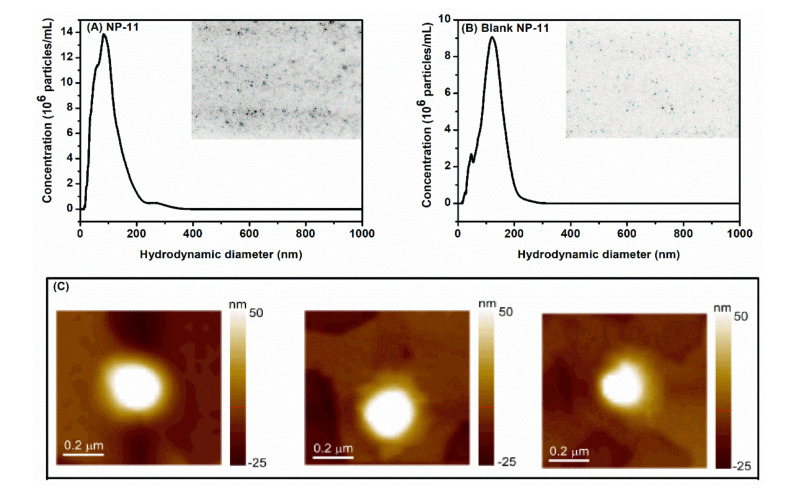
Size distribution for (**A**) NP-11 and (**B**) blank NP-11 from NTA measurements. Inserts show NTA video frames for both samples. (**C**) AFM images obtained for NP-11.

The NTA was performed complementary to multiangle DLS since it detects small and weakly scattering particles, even in the presence of large and strong scatterers, particles, and/or agglomerates [40,41]. Figure 4A,B displays the NTA results for NP-11 and blank NP-11, respectively (the inserts show the NTA video frame of the light scattered by particles moving under Brownian motion). The particle size distributions for NP-11 and blank NP-11 nanoparticles showed a narrow peak, indicating a relatively monodisperse sample, with a peak at 84 and 121 nm in diameter, respectively. The small difference in the hydrodynamic diameter obtained by NTA and multiangle DLS measurements can be explained considering that size distributions obtained by the former consist of number distributions, while that obtained from the latter are intensity distributions [42]. Furthermore, AFM images presented in Figure 4C helped to highlight MH-b-PS@TMC nanoparticles’ morphology. Nanoparticles showed an almost spherical shape with diameters similar to those determined by both DLS and NTA techniques.

### 2.2. Encapsulation Efficiency, Drug Loading, and In Vitro Release Kinetics by the HPLC-UV Method

The HPLC–UV method was suitable for quantification of drugs loaded in MH-b-PS nanoparticles, and to evaluate the in vitro drug release kinetic. The results regarding to the method such as linearity, coefficient of determination (R^2^), selectivity and sensitivity (limit of detection (LOD), and quantification (LOQ)) are available in Supplementary Material. Many factors may affect the drug content, such as polarity and nature of the active substance, its solubility in the core of the nanoparticles, and the type of oil used. NP-11 showed a drug content of 238.6 ± 6.8 µg mL^−1^ with an encapsulation efficiency of 80.9 ± 0.4 %. Considering these results, it can be inferred that the MH-b-PS nanoparticle formulation presents appropriate anticancer drug content for its use towards human breast carcinoma.

**Figure 5:**
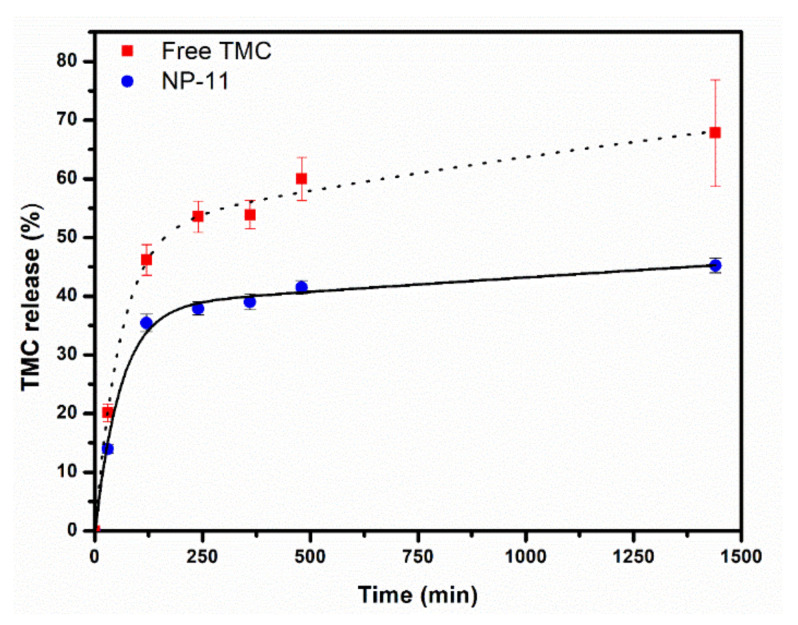
In vitro release profile for free TMC and NP-11 using the dialysis bag method. The solid line corresponds to biexponential fitting for NP-11 and the dotted line is solely a guide in respect to free TMC.

In vitro release of TMC from NP-11 was carried out using the dialysis bag method, under sink conditions, using simulated intestinal fluid as the release medium to simulate the organism’s conditions. For comparison purposes, the release of free TMC was also investigated under the same conditions. Figure 5 shows the cumulative percentage of drug release profile from NP-11 and for free TMC, during a period of 24 h. As expected, free TMC displays a faster release than MH-*b*-PS@TMC nanoparticles, and the amount released was 68% up to 24 h. On the other hand, NP-11 exhibited a drug release of 45% in the same time interval. The lowest percentage of drug release from NP-11 with respect to free TMC may be explained considering that several factors affect the release rate from nanoparticles by dynamic dialysis: (i) the effective drug concentration within the nanoparticle; (ii) the rate constants for permeation across the nanocarrier and dialysis membrane; and (iii) effective drug concentration within the dialysis bag once the drug is released from nanoparticles [43]. Furthermore, it should be kept in mind that the drug transport from polymeric nanoparticles may occur through complex mechanisms such as diffusion through pores or polymer matrix, the osmotic effect, and surface erosion [44]. As can be seen in Table 2, among the different mathematical models used to evaluate the drug release profile from NP-11, the biexponential model [43,45] better fits the data (R^2^ close to 1) (see also fit in Figure 5). This model indicates fast release at an early stage, followed by a sustained release profile governed by slow drug diffusion through MH-b-PS nanoparticles. The drug release profile observed for NP-11 is a classical biphasic model, consisting of rapid release (phase I), referred to as the burst effect, followed by slow drug release (phase II) [44,46]. The burst release observed for NP-11 in the early stage may be attributed to TMC adsorbed on the nanoparticle surface, evidenced also by the positive ζ potential observed for the nanoparticles containing the drug. Interestingly, a similar initial burst release was observed for other tamoxifen-loaded nanoparticles, such as poly(ε-caprolactone) [47], chitosan-gellan [48], and poly(ethylene glycol)-modified cyanoacrylate nanoparticles [49]. Analyzing the values of the kinetic parameters of the biexponential model, it can be seen that the rate constant in the phase II ($k_{2}\left(min^{-1}\right)$) is much smaller than phase I ($k_{1}\left(min^{-1}\right)$), highlighting that MH-b-PS@TMC nanoparticles are a promising sustained-release drug delivery system for cancer therapy. Furthermore, the coefficients a and b of the biexponential model indicate that up to 24 h, the amount of TMC released from NP-11 was 38% in the early stage (a) and 62% in the sustained stage (b). These outcomes suggest that most of the anticancer drug was entrapped inside the hydrophobic core of NP-11 being released slowly in the phase II: this means that formulation of TMC into nanoparticles could potentially lead to a longer and sustained release of the drug, potentially reducing side-effects and frequency of administration of the therapy.

### 2.3. Cell Culture Studies

**Figure 6:**
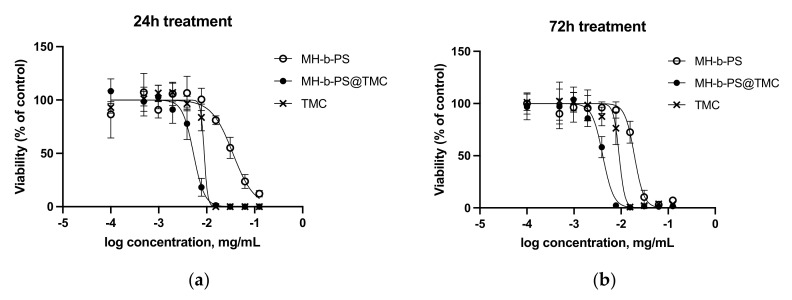
Cytotoxicity for blank MH-*b*-PS nanoparticles (empty circle), MH-*b*-PS@TMC (full circle), and TMC solution (cross) on the MCF-7 cell line, as estimated by MTT assay (**a**) after 24 h or (**b**) 72 h of incubation.

MCF-7 cells were exposed, respectively, to MH-*b*-PS@TMC (NP-11), blank MH-b-PS nanoparticles (blank NP-11), or TMC solution in serum-free MEM. Graphs representing cell viability after 24 or 72 h of exposure to treatments are reported in Figure 6a,b respectively, and related IC_50_ are listed in Table 3.

For the TMC solution, the same value for IC_50_ was estimated at both timepoints, while for MH-b-PS and MH-Bb-PS@TMC, a reduction of IC_50_ was observed in time. This is a re-markable result indicating that targeted and sustained delivery of the drug occurs that is imputable to the presence of the nanosized vehicle. IC_50_ for MH-b-PS@TMC was at least half the value of IC_50_ for the TMC solution, suggesting that the vehicle could potentially reduce the dose that is necessary to get the same lethal effect. This could be attributed to a more efficient intracellular delivery of the drug, probably due to the binding and subsequent internalization of nanoparticles by cells. Binding of nanoparticles could be mediated by interaction of maltoheptaose corona with cell receptors promoting MH-b-PS@TMC internalization by cells, which could be endocytosis-dependent. It is presumable that the overexpression of the glucose transporter family [50] and in particular of glucose transporter 1, GLUT 1, by the MCF-7 cell line could provide a preferential entry route for maltoheptaose-coated nanoparticles, as previously observed also for glucose-modified PAMAM-based dendrimers loaded with doxorubicin [51]. This could result in selective toxicity of MH-b-PS@TMC against highly proliferating cells, with respect to non-cancerous tissues, further improving their therapeutic index with respect to free TMC. Moreover, in MH-b-PS@TMC, the dissolution of the drug in the oil phase could be a further element contributing to its prompt availability. At corresponding concentrations of blank MH-b-PS nanoparticles, no toxicity was observed, excluding that increased cytotoxicity observed for drug-loaded nanoparticles could be ascribed to the polymer or to the delivery system as such, which shows no toxic effects at all on MCF-7 viability up to concentrations three times higher than the estimated IC_50_ for MH-b-PS@TMC. No conventional surfactants were needed for the preparation of nanoparticles, excluding a potential source of toxicity, while other components of the vehicle possess a good safety profile. Further studies will provide a deeper comprehension of the mechanism of delivery and confirm that no long-term toxicity can be ascribed to the vehicle.

## 3. Materials and Methods

### 3.1. Materials

Tamoxifen citrate (TMC) was kindly provided by Lisapharma S.p.A. (Erba, Italy). Labrafac Lipophile WL 1349 (medium chain triglyceride) was supplied by Gattefossé SAS (Saint-Priest, France). Tetrahydrofuran (Chromanorm for HPLC, purity > 99.7%) and acetonitrile (HPLC grade) were purchased from VWR Chemicals (Milan, Italy). Sodium phosphate monobasic (NaH_2_PO_4_) was purchased from Sigma Aldrich (St. Louis, MO, USA). Ultrapure water (conductivity less than 0.05 μS·cm^−1^) was obtained from a Milli-Q water system (Millipore^®^, Billerica, MA, USA), filtered through 0.22 μm hydrophilic filters (Sartorius, Barcelona, Spain) and used to prepare the solutions.

The MH_1_._2k_-b-PS_4_._5k_ (subscript denotes the molecular weight of the corresponding block) was synthesized by “click” reaction between propargyl-functionalized MH (propargyl-MH_1_._2k_) and azido end-functionalized PS (PS_4_._5k_-N_3_) in DMF as solvent, in the presence of a Cu catalyst, according to a previous report [19]. The scheme of MH-b-PS synthesis is available in the Supporting Information (SI) (see Figure S1).

Cell line MCF-7 was obtained from the American Type Culture Collection (HTB-22, Manassas, VA, USA). Cell culture medium MEM (Minimum Essential Medium), Fetal Bovine Serum (FBS), non-essential amino acids solution (NEAA), and HBSS were supplied by Gibco Life Technology (Thermo Fisher Scientific, Monza, Italy), and penicillin-streptomycin solution was supplied by Aurogene (Rome, Italy). MTT (3-(4,5-dimethylthiazol-2-yl)-2,5-dyphenyltetrazolium bromide) reagent and HEPES buffer were purchased from Sigma-Aldrich (St. Louis, MO, USA). Dulbecco’s phosphate buffered saline without Ca^2+^ and Mg^2+^ (DPBS) was provided by Corning (Corning, New York, USA). For cell cultivation, 75 cm^2^ vented flasks were from Nunc (Thermo Fisher Scientific, Monza, Italy) while 96-well plates were from VWR International (Milan, Italy).

### 3.2. Nanoparticles Preparation

The MH-b-PS@TMC nanocapsules were prepared by a nanoprecipitation method previously described in the literature, with some modifications [16]. Six formulations containing MH-b-PS copolymer, TMC, and Labrafac oil were prepared using the reverse nanoprecipitation method and five formulations were prepared using the standard nanoprecipitation method. A detailed protocol for preparation of nanoparticles and the compositions of formulations (see Tables S1 and S2) are presented in the SI. Briefly, a well-defined amount of MH-b-PS copolymer was dispersed in THF/water mixture using a magnetic stirring bare (500 rpm) for a specific time, under temperature control. The dispersion was added to a flask containing TMC and Labrafac^®^ oil, and left under stirring (500 rpm) for 30 min, at 25 °C. Then, the nanoparticles were prepared using two different protocols. For reverse nanoprecipitation, a well-defined amount of milli-Q water was slowly added dropwise with a Pasteur pipette to dispersion (copolymer, drug and oil) and left under stirring (500 rpm) during 2 h at 25 °C. For standard nanoprecipitation, a well-defined amount of dispersion (copolymer, drug and oil) was slowly added dropwise with a Pasteur pipette to 40 g of milli-Q water and left under stirring (500 rpm) during 2 h at 25 °C. Subsequently, the dispersions obtained from both methods were concentrated until the final mass of 4 g by evaporation under reduced pressure at 39 °C ± 1 °C.

### 3.3. Physicochemical Characterization of MH-b-PS Nanoparticles

The hydrodynamic diameter (D_h_), polydispersity index (PDI) and ζ potential of all nanoparticles were measured using a Nanozetasizer ZS (Nano ZS Zetasizer Nanoseries, Malvern Instruments, Worcestershire, UK), which operates with a laser source of He–Ne (λ = 633 nm and P = 5 mW). The scattered light was measured at a fixed angle (θ) of 173° and the samples were thermostatized at 25 °C. The D_h_ and PDI of the nanoparticles were measured in triplicate (*n* = 3) without dilution and analyses were carried out 10 times for each sample. The Smoluchowski approximation was used to calculate the ζ potential and the measurements (20 runs for each measure) were performed in triplicate (*n* = 3).

The particle size of the nanocapsules formulation chosen for tamoxifen loading was evaluated also by multi-angle light scattering technique, nanoparticle tracking analysis (NTA), and atomic force microscopy (AFM). The multi-angle light scattering experiments were carried out at 25 °C using an ALV laser goniometer (Langen, Germany), which consists of a 22 mW HeNe linearly polarized laser operating at a wavelength of 632.8 nm and an ALV-5000/EPP multiple τ digital correlator with 125 ns initial sampling time. The light scattering intensity was measured at different angles in the range of 40° to 130°, with steps of 10°. The light scattering autocorrelation functions were analyzed by means of constrained regularization (CONTIN) to obtain the distribution of decay times and their corresponding amplitude. From the linear dependence of the relaxation frequency ($\Gamma \left(s^{-1}\right)$) on the square of wave vector modulus (q^2^), the diffusion coefficient (D) and hydrodynamic radius (R_h_) of the nanoparticle was determined.

NTA experiments were performed using a LM10 system (NanoSight, Salisbury, UK). The NanoSight instrument involves particle-tracking software (NTA version 2.1) accompanied by microscopy setup and a charge-coupled device (CCD) camera that allows for visualization and tracking of laser (488 nm) illuminated particles undergoing Brownian motion. The nanoparticles dispersions were directly put into the chamber using a sterile syringe until the liquid reached the tip of the nozzle. The instrument software was used to capture and analyze the video sequences captured over 60 s.

Atomic force microscopy (AFM) measurements were performed by deposition of MH-b-PS@TMC nanoparticles on a mica plate and allowed to evaporate at room temperature. The samples were scanned in intermittent contact and non-contact modes. Topographic images were taken using a Park NX10 microscope (Park Systems, Suwon, South Korea) equipped with Smart Scan software version 1.0.RTM11a. The measurements were performed with a highly doped silicon monolithic probe with a reflective aluminum coating (PPP-NCHR, Nanosensors, Neuchâtel, Switzerland) with a nominal resonance frequency of 320 kHz and a constant force of 42 N/m. All measurements were performed at a temperature of 25 ± 0.5 °C and relative humidity of 55 ± 10%, with a scanning rate between 0.35 and 1.00 Hz. The treatment of the images was done with the XEI software version 4.3.4Build22.RTM1. For statistical representativeness, each sample had images obtained in three different regions.

### 3.4. Determination of Drug Content and Encapsulation Efficiency

The drug content was determined in triplicate after dissolution of MH-b-PS@TMC nanoparticle in the mixture acetonitrile/methanol (50:50 *v*/*v*): 200 μL of nanoparticle were dissolved in the mixture to bring to a final volume of 10 mL. The sample was subjected to magnetic stirring for 30 min at 25 °C, sonicated for 30 min using an ultrasonic bath and followed by filtration in a 0.45 μm regenerated cellulose membrane filter (Sartorius, Varedo, Italy). The drug content in the nanoparticles was quantified using a validated HPLC method [10]. A Shimadzu HPLC apparatus (Kyoto, Japan), equipped with Symmetry Shield RP8 Column (100 Å, 5 µm, 3.9 × 150 mm, Waters Corporation, Sesto San Giovanni, Italy), was used. The mobile phase was a mixture (40:60) of acetonitrile:phosphate buffer containing 0.9 g·L^−1^ of sodium dihydrogen phosphate, adjusted to pH 3.0 with phosphoric acid. Flow rate was set at 1.0 mL·min^−1^ and injection volume was 100 μL. UV detection was performed at 240 nm and the column oven temperature was set at 30 °C. Validation of the HPLC assay were demonstrated under optimized conditions regarding its linearity range, coefficient of determination (R^2^), selectivity and sensitivity (limit of detection (LOD), and limit of quantification (LOQ), according to bioanalytical methods. Free drug (FC) was determined in the ultrafiltrate after separation of the nanoparticles (*n* = 3) by ultrafiltration/centrifugation (Amicon^®^ Ultra 0.5 mL, MW 3 kDa, Millipore, Carrigtwohill, Ireland) at 3000 rpm for 20 min. The drug in the supernatant was measured using a calibration curve through HPLC. The encapsulation efficiency (EE) was calculated by the difference between the drug content (DC) and free drug (FC) determined in the nanoparticles, according to Equation (1):

(1) $$EE\left(\%\right)=\left(\frac{DC-FC}{DC}\right)*100$$

### 3.5. In Vitro Release Kinetics

In vitro release profile of TMC loaded MH-b-PS nanoparticles was performed using the dialysis bag method [48]. The release experiments were carried out using the simulated intestinal fluid (SIF) (pH 7.4) as the release medium (external medium). Initially, drug solubility studies were performed in the release medium to ensure sinking conditions. In a typical experiment, 1.0 mL aliquots of the nanoparticle containing 250 µg·mL^−1^ of TMC were placed in dialysis bags (molecular weight cut-off of 14,000 Da, cellulose membrane, average flat width of 33 mm, Sigma Aldrich, St. Louis, MO, USA) and incubated in 25 mL of the release medium. The system was kept under constant and moderate magnetic stirring at 37 °C. At fixed time intervals up to 24 h, 1.0 mL aliquots were removed from the release medium for the quantification of TMC by the HPLC method. After removing the aliquot, 1.0 mL of the release medium was added to the recipient to maintain a constant volume. For comparison, the release kinetics of free TMC (non-encapsulated) was also investigated using the same experimental set-up reported above. The experiments were carried out in triplicate (*n* = 3) and the results are presented as the percentage (%) of drug released as a function of time. To evaluate the drug release profile from NP-11, the data were fitted using six kinetic models, including zero order, first order, biexponential, Higuchi, Korsmeyer–Peppas, and Hixon Crowell [46,52,53].

### 3.6. In Vitro Cytotoxicity on MCF-7 Cells

The tamoxifen-sensitive human breast cancer cell line MCF-7 was grown and expanded in growth medium composed of MEM medium supplemented with 10% FBS, 1× non-essential amino acids solution and 1× penicillin-streptomycin solution in 75 cm^2^ culture flasks and incubated at 37 °C in 5% CO_2_ atmosphere. Cells were split weekly (1:2) by detaching them with 0.05% trypsin-EDTA solution and seeded for toxicity assays in 96 well plates at a density of 2·10^4^ cells/well in 100 µL of growth medium. After overnight incubation, culture medium was removed, cells were washed with DPBS, and exposed to tamoxifen-loaded NPs or tamoxifen citrate solution diluted in serum free-MEM, in a geometric series dilution at a ratio equal to 2. Nine concentration levels were assayed with respect to corresponding amounts of unloaded nanoparticles or a control without the drug. To exclude effects on cell viability due to unloaded NPs or medium dilution, toxicity of each sample was expressed with respect to cell survival in a drug-free medium at same dilutions.

After 72 h of incubation at 37 °C, 5% CO_2_, cell viability was measured in terms of mitochondrial activity by using MTT assay. Test formulations were gently removed and 150 µL of 1 mg·mL^−1^ solution of thyazol blue tetrazolium bromide in HBSS + 30 mM HEPES were added and left for 2 h (37 °C, 5% CO_2_). Formazan crystals formed and those remaining in wells after having removed the MTT solution were dissolved in 120 μL of DMSO for each well, under shaking, for 10 min in the dark. Absorbance of samples was then read at 570 nm by means of a plate reader (Spark^®^ Tecan, Mannedorf, Switzerland) and viability expressed as a percentage with respect to the untreated control cells. Inhibitory concentration (IC_50_) was estimated by means of Prism9 (GraphPad Software, San Diego, CA, USA) on a non-linear fitting of % viability vs log_10_ concentration. TMC loaded MH-b-PS nanoparticles presented in this work have the potential to widen the therapeutic arsenal for the treatment and prevention of breast cancer. The formulations prepared by standard nanoprecipitation proved to be better than the reverse method for obtaining nanoparticles with small size and narrow particle size distribution. The MH-b-PS@TMC nanoparticles presented suitable conditions for their use in cancer therapy since they are stable and can encapsulate a high amount of drug (high drug content and encapsulation efficiency) without the need for conventional surfactants. The in vitro drug release profile of TMC was best described by a biphasic model, indicating a fast release at early time points, followed by a sustained release controlled by diffusion. The MH-b-PS@TMC nanoparticles showed higher cytotoxicity towards MCF-7 cells with respect to free tamoxifen citrate that could not be attributed to nanoparticles themselves, but rather confirms the effectiveness of loaded nanoparticles in delivering the drug to target cells, probably by means of internalization mediated by glucose transporters. Further studies will investigate the role of the sugar block of maltoheptaose on the mechanism of cell uptake with a view to oral administration, including evaluation of nanoparticle behavior in gastric fluid and evaluation of absorption by an in vitro model of intestinal epithelium, such as the Caco-2 cell line.

## Figures and Tables

**Table 1:** Physicochemical properties of MH-b-PS@TMC nanoparticles.

Sample	D_h_ (nm) ^1^	PDI ^2^	ζ Potential (mV)
Reverse Nanoprecipitation			
NP-1	243 ± 2.3	0.270 ± 0.006	+31.53 ± 0.21
NP-2	300 ± 8.2	0.474 ± 0.068	+36.90 ± 0.42
NP-3	229 ± 0.8	0.197 ± 0.010	+26.40 ± 0.07
NP-4	277 ± 20.3	0.373 ± 0.032	+37.97 ± 0.61
NP-5	264 ± 4.3	0.246 ± 0.010	+32.16 ± 0.89
NP-6	438 ± 5.6	0.292 ± 0.004	+24.10 ± 0.46
Standard Nanoprecipitation			
NP-7	82 ± 0.6	0.119 ± 0.014	+20.70 ± 1.99
NP-8	74 ± 0.6	0.146 ± 0.003	+21.53 ± 1.20
NP-9	74 ± 0.5	0.145 ± 0.006	+21.90 ± 3.30
NP-10	78 ± 0.6	0.113 ± 0.015	+20.80 ± 0.50
NP-11	74 ± 2.5	0.118 ± 0.004	+21.50 ± 3.00
Blank NP-11	88 ± 7.1	0.094 ± 0.032	−11.73 ± 3.69

^1^ Hydrodynamic Diameter ($D_{h}=2R_{h})$; ^2^ Polydispersity index.

**Table 2:** Rate constants $\left(k\right)$ and coefficient of determination ($R^{2})$ obtained for various mathematical models of drug release.

Model		Parameters	
	$k_{1}$	$k_{2}$	$R^{2}$
Zero Order	0.11		0.8299
First Order	0.01		0.7157
Biexponential	$1.70\cdot 10^{-2}$ a=0.38	$8.47\cdot 10^{-5}$ b=0.62	0.9960
Higuchi	2.19		0.9609
Korsmeyer–Peppas	7.12 n=0.29		0.9437
Hixon–Crowell	0.0096		0.7319

**Table 3:** Comparison of IC_50_ for blank MH-b-PS nanoparticles, MH-B-PS@TMC, and TMC solution after 24 h or 72 h of application. The data represent mean and 95% confidence interval (CI), *n* = 6.

Treatment	IC_50_ @ 24 h (µg/mL)	IC_50_ @ 72 h (µg/mL)
Blank MH-*b*-PS	39.43 (30.64–51.03)	19.20 (14.85–24.86)
MH-*b*-PS@TMC	5.14 (3.96–6.68)	3.85 (2.98–4.97)
TMC solution	9.95 (7.32–13.54)	9.00 (6.81–11.98)

## Data Availability

Data are contained within the article.

## Supplementary Materials

Figure S1: Scheme of the synthesis of MH1.2k-b-PS4.5k. Figure S2: Calibration standard curve for TMC. Insert: HPLC chromatogram of TMC. Figure S3: HPLC chromatogram for nanoparticle without drug (blank NP-11). Table S1: Composition of the Formulations and Final Concentration of Reagents for Nanoparticles obtained by Reverse Nanoprecipitation Method. Table S2: Composition of the Formulations and Final Concentration of Reagents for Nanoparticles obtained by Standard Nanoprecipitation Method. Text: Protocol for preparation of MH-b-PS@TMC nanoparticles and HPLC-UV Results.

## Sample Availability

Samples of the compounds are not available from the authors.
