# Supplementary material for: Design and Characterization of Maltoheptaose-b-Polystyrene Nanoparticles, as a Potential New Nanocarrier for Oral Delivery of Tamoxifen
Source: Molecules. 2021 Oct 28;26(21):6507. doi: 10.3390/molecules26216507 (PMC8587208; doi:10.3390/molecules26216507)
Supplement: Supplementary file 1 [file molecules-26-06507-s001.zip › molecules-1417236-supplementary.pdf]

Supplementary Materials

Design and Characterization of Maltoheptaose-b-Polystyrene Nanoparticles, as a Potential New Nanocarrier for Oral Delivery of Tamoxifen

Marcos Antonio Villetti [1], Adryana Clementino [2,], Ilaria Dotti [3], Patricia Ebani [1], Eride Quarta [3], Francesca Buttini [2,3], Fabio Sonvico [2,3], Annalisa Bianchera [2,3,*] and Redouane Borsali[4,*]

[1] Laboratório de Espectroscopia e Polímeros (Lepol), Departamento de Física, Universidade Federal de Santa Maria, Santa Maria (RS), Brazil; mvilletti@ufsm.br; patricia.ebani@ufsm.br

[2] Biopharmanet-TEC, University of Parma, 43124 Parma, Italy; adryana.rochaclementino@studenti.unipr.it

[3] Department of Food and Drug, University of Parma, 43124 Parma, Italy; ilaria.dotti@studenti.unipr.it; eride.quarta@studenti.unipr.it; francesca.buttini@unipr.it; fabio.sonvico@unipr.it; annalisa.bianchera@unipr.it

[4] Univ. Grenoble Alpes, CNRS, CERMAV, 38000 Grenoble, France, E-mail: borsali@cermav.cnrs.fr

* Correspondence: annalisa.bianchera@unipr.it ; borsali@cermav.cnrs.fr

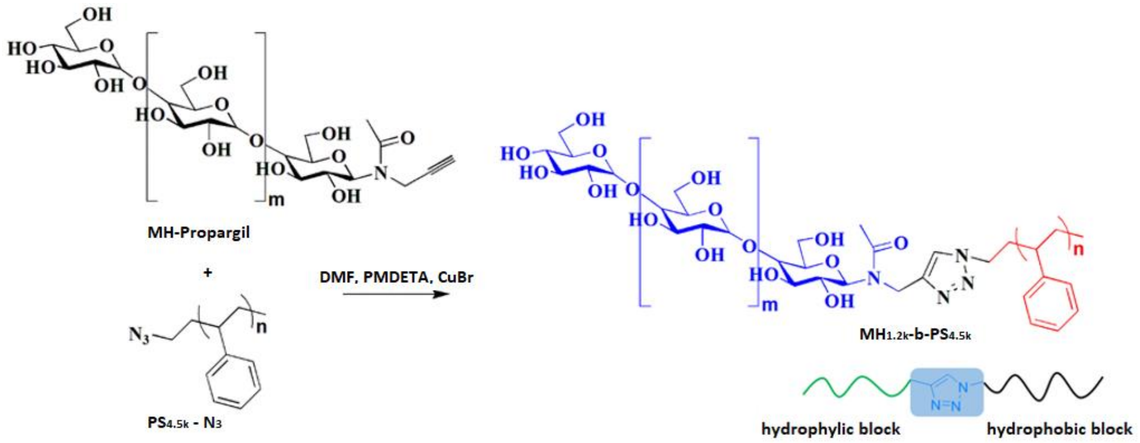

Figure S1. Scheme of the synthesis of $MH_{1.2k}$-b-$PS_{4.5k}$.

Table S1. Composition of the Formulations and Final Concentration of Reagents (mg/g) for Nanoparticles obtained by Reverse Nanoprecipitation Method.

	MH-b-PS (mg)	THF/H_2O (8:2 w/w) (g)	time/T(°C) dissolution copolymer	m_{TMC} (mg)	m_{Oil} (mg)	mdispersion# (g)	[MH-b-PS] (mg/g)	[TMC] (mg/g)	[Oil] (mg/g)
Copolymer + TMC + Oil									
**NP-1**	1.0	1.0	2h/25°C	1.0	1.5	1.0	0.250	0.250	0.375
**NP-2**	2.0	1.0	2h/25°C	2.0	2.9	1.0	0.500	0.500	0.735
**NP-3**	1.0	1.0	2h/25°C**	1.0	1.4	1.0	0.250	0.250	0.350
**NP-4**	2.0	1.0	2h/25°C**	2.0	2.6	1.0	0.500	0.500	0.65
**NP-5**	3.0	2.0	2h/25°C**	3.0	4.2	1.0	0.375	0.375	0.525
**NP-6**	2.0	2.0	2h/25°C**	2.0	2.8	1.0	0.250	0.250	0.350

$m_{dispersion}$ = mass of the dispersion (copolymer + THF + water) used in the formulation to prepare the nanoparticles.

** The dispersion was placed on the ultrasound for 15 min before the rotary evaporation (for each 5 min on the ultrasound one minute at rest).

Table S2. Composition of the Formulations and Final Concentration of Reagents (mg/g) for Nanoparticles obtained by Standard Nanoprecipitation Method.

	MH-b-PS (mg)	THF/H_2O (8:2 w/w) (g)	time/T(°C) dissolution copolymer	m_{TMC} (mg)	m_{Oil} (mg)	mdispersion# (g)	[MH-b-PS] (mg/g)	[TMC] (mg/g)	[Oil] (mg/g)
Copolymer + TMC + Oil									
**NP-7**	1.0	1.0	2h/25°C *	1.0	1.4	1.0	0.250	0.25	0.35
**NP-8**	3.0	3.0	3h/25°C **	1.0	1.4	1.0	0.250	0.25	0.35
**NP-9**	3.0	3.0	3h /40°C ***	1.0	1.4	1.0	0.250	0.25	0.35
**NP-10**	3.0	3.6	3h /40°C ***	1.0	1.4	1.8	0.375	0.25	0.35
**NP-11**	3.0	3.6	3h/40°C	1.0	1.4	1.8	0.375	0.25	0.35
Copolymer + Oil									
**Blank NP-11**	3.0	3.6	3h/40°C	-	1.4	1.8	0.375	-	0.35

# $m_{dispersion}$ = mass of the dispersion (copolymer + THF + water) used in the formulation to prepare the nanoparticles.

* The dispersion was placed on the ultrasound for 15 min before the rotary evaporation (for each 5 min on the ultrasound one minute at rest).

** The dispersion was placed on the ultrasound for 15 min after dissolution of the copolymer in THF/water mixture (for each 5 min on the ultrasound one minute at rest).

*** The dispersion was placed on the ultrasound for 15 min before its addition to 40 g of milli-Q water (for each 5 min on the ultrasound one minute at rest).

Protocol for preparation of MH-b-PS@TMC nanoparticles

A) Reverse nanoprecipitation method

A.1) NP-1. First, the MH-b-PS (1 mg) was dispersed in 1 g of THF/water mixture (8:2 w/w) using a magnetic stirring bar (500 rpm) during 2 h, at 25 °C. After that, the dispersion was added to a flask containing 1 mg of TMC and 1.4 mg of oil, and left under sitting (500 rpm) during 30 min, at 25 °C. Then, 40 g of milli-Q water was added dropwise with a Pasteur pipette to 1 g of dispersion (copolymer, the drug and the oil) and left under stirring (500 rpm) during 2 h at 25 °C. Finally, the dispersion was rotoevaporated under reduced pressure at temperature of 39 ± 1 °C, until a final mass of 4 g.

A.2) NP-2. First, the MH-b-PS (2 mg) was dispersed in 1 g of THF/water mixture (8:2 w/w) using a magnetic stirring bar (500 rpm) during 2 h, at 25 °C. After that, the dispersion was added to a flask containing 2 mg of TMC and 2.9 mg of oil, and left under sitting (500 rpm) during 30 min, at 25 °C. Then, 40 g of milli-Q water was added dropwise with a Pasteur pipette to 1 g of dispersion (copolymer, the drug and the oil) and left under

stirring (500 rpm) during 2 h at 25 °C. Finally, the dispersion was rotoevaporated under reduced pressure at temperature of 39 ± 1 °C, until a final mass of 4 g.

A.3) NP-3. First, the MH-b-PS (1 mg) was dispersed in 1 g of THF/water mixture (8:2 w/w) using a magnetic stirring bar (500 rpm) during 2 h, at 25 °C. After that, the dispersion was added to a flask containing 1 mg of TMC and 1.4 mg of oil, and left under sitting (500 rpm) during 30 min, at 25 °C. Then, 40 g of milli-Q water was added dropwise with a Pasteur pipette to 1 g of dispersion (copolymer, the drug and the oil) and left under stirring (500 rpm) during 2 h at 25 °C. The dispersion was added to an erlenmeyer flask with lid and sonicated (Ultrasound VWR USC300T; frequency 45 kHz and power 80 W) during 15 min (at each 5 min sonification 1 min of resting). After sonification, the dispersion was rotoevaporated under reduced pressure at temperature of 39 ± 1 °C, until a final mass of 4 g.

A.4) NP-4. First, the MH-b-PS (2 mg) was dispersed in 1 g of THF/water mixture (8:2 w/w) using a magnetic stirring bar (500 rpm) during 2 h, at 25 °C. After that, the dispersion was added to a flask containing 2.0 mg of TMC and 2.6 mg of oil, and left under sitting (500 rpm) during 30 min, at 25 °C. Then, 40 g of milli-Q water was added dropwise with a Pasteur pipette to 1 g of dispersion (copolymer, the drug and the oil) and left under stirring (500 rpm) during 2 h, at 25 °C. The dispersion was added to an erlenmeyer flask with lid and sonicated (Ultrasound VWR USC300T; frequency 45 kHz and power 80 W) during 15 min (at each 5 min sonification 1 min of resting). After sonication, the dispersion was rotoevaporated under reduced pressure at temperature of 39 ± 1 °C, until a final mass of 4 g.

A.5) NP-5. First, the MH-b-PS (3 mg) was dispersed in 2 g of THF/water mixture (8:2 w/w) using a magnetic stirring bar (500 rpm) during 2 h, at 25 °C. After that, the dispersion was added to a flask containing 3 mg of TMC and 4.2 mg of oil, and left under sitting (500 rpm) during 30 min, at 25 °C. Then, 40 g of milli-Q water was added dropwise with a Pasteur pipette to 1 g of dispersion (copolymer, the drug and the oil) and left under stirring (500 rpm) during 2 h at 25 °C. The dispersion was added to an erlenmeyer flask with lid and sonicated (Ultrasound VWR USC300T; frequency 45 kHz and power 80 W) during 15 min (at each 5 min sonification 1 min of resting). After sonication, the dispersion was rotoevaporated under reduced pressure at temperature of 39 ± 1 °C, until a final mass of 4 g.

A.6) NP-6. First, the MH-b-PS (2 mg) was dispersed in 2 g of THF/water mixture (8:2 w/w) using a magnetic stirring bar (500 rpm) during 2 h, at 25 °C. After that, the dispersion was added to a flask containing 2 mg of TMC and 2.8 mg of oil, and left under sitting (500 rpm) during 30 min, at 25 °C. Then, 40 g of milli-Q water was added dropwise with a Pasteur pipette to 1 g of dispersion (copolymer, the drug and the oil) and left under stirring (500 rpm) during 2 h at 25 °C. The dispersion was added to an erlenmeyer flask with lid and sonicated (Ultrasound VWR USC300T; frequency 45 kHz and power 80 W) during 15 min (at each 5 min sonification 1 min of resting). After sonication, the dispersion was rotoevaporated under reduced pressure at temperature of 39 ± 1 °C, until a final mass of 4 g.

B) Standard nanoprecipitation method

B.1) NP-7. First, the MH-b-PS (1 mg) was dispersed in 1 g of THF/water mixture (8:2 w/w) using a magnetic stirring bar (500 rpm) during 2 h, at 25° C. After that, the dispersion was added to a flask containing 1 mg of TMC and 1.4 mg of oil, and left under sitting (500 rpm) during 30 min, at 25 °C. Then, 1 g of dispersion (copolymer, the drug and the oil) was added dropwise with a Pasteur pipette to 40 g of milli-Q and left under stirring (500 rpm) during 2 h, at 25 °C. The dispersion was added to an erlenmeyer flask with lid and sonicated (Ultrasound VWR USC300T; frequency 45 kHz and power 80 W) during 15 min (at each 5 min sonification 1 min of resting). After sonication, the dispersion was rotoevaporated under reduced pressure at temperature of 39 ± 1 °C, until a final mass of 4 g.

B.2) NP-8. First, the MH-b-PS (3 mg) was dispersed in 3 g of THF/water mixture (8:2 w/w) using a magnetic stirring bar (500 rpm) during 3 h, at 25° C. The dispersion was sonicated (Ultrasound VWR USC300T; frequency 45 kHz and power 80 W) during 15 min (at each 5 min sonification 1 min of resting). After that, 1 g of dispersion was added to a flask containing 1 mg of TMC and 1.4 mg of oil and left under sitting (500 rpm) during 30 min, at 25° C. Then, 1 g of dispersion (copolymer, the drug and the oil) was added dropwise with a Pasteur pipette to 40 g of milli-Q and left under stirring (500 rpm) during 2 h, at 25 °C. Finally, the dispersion was rotoevaporated under reduced pressure at temperature of 39 ± 1 °C, until a final mass of 4 g.

B.3) NP-9. First, the MH-b-PS (3 mg) was dispersed in 3.0 g of THF/water mixture (8:2 w/w) using a magnetic stirring bar (500 rpm) during 3 h, at 40° C. After that, 1 g of dispersion was slowly added to a flask containing 1 mg of TMC and 1.4 mg of oil and left under magnetic stirring (500 rpm) during 30 min, at 25° C. Then, the dispersion was

sonicated (Ultrasound VWR USC300T; frequency 45 kHz and power 80 W) during 15 min (at each 5 min sonification 1 min of resting). After that, 1 g of dispersion (copolymer, the drug and the oil) was added dropwise with a Pasteur pipette to 40 g of milli-Q and left under stirring (500 rpm) during 2 h, at 25 °C. Finally, the dispersion was rotoevaporated under reduced pressure at temperature of 39 ± 1 °C, until a final mass of 4 g.

B.4) NP-10. First, the MH-b-PS (3 mg) was dispersed in 3.6 g of THF/water mixture (8:2 w/w) using a magnetic stirring bar (500 rpm) during 3 h, at 40° C. After that, 1.8 g of the dispersion slowly added to a flask containing 1 mg of TMC and 1.4 mg of oil, and left under magnetic stirring (500 rpm) during 30 min at 25° C. Then, the dispersion was sonicated (Ultrasound VWR USC300T; frequency 45 kHz and power 80 W) during 15 min (at each 5 min sonification 1 min of resting). After that, 1.8 g of dispersion (copolymer, drug and oil) was added dropwise with a Pasteur pipette to 40 g of milli-Q water and left under stirring (500 rpm) during 2 h, at 25 °C. Finally, the dispersion was rotoevaporated under reduced pressure at temperature of 39 ± 1 °C, until a final mass of 4 g.

B.5) NP-11. First, the MH-b-PS (3 mg) was dispersed in 3.6 g of THF/water mixture (8:2 w/w) using a magnetic stirring bar (500 rpm) during 3 h, at 40° C. After that, 1.8 g of the dispersion slowly added to a flask containing 1 mg of TMC and 1.4 mg of oil, and left under magnetic stirring (500 rpm) during 30 min at 25° C. Then, 1.8 g of dispersion (copolymer, drug and oil) was added dropwise with a Pasteur pipette to 40 g of milli-Q water and left under stirring (500 rpm) during 2 h, at 25 °C. Finally, the dispersion was rotoevaporated under reduced pressure at temperature of 39 ± 1 °C, until a final mass of 4 g.

B.6) Blank NP-11. First, the MH-b-PS (3 mg) was dispersed in 3.6 g of THF/water mixture (8:2 w/w) using a magnetic stirring bar (500 rpm) during 3 h, at 40° C. After that, 1.8 g of the dispersion slowly added to a flask containing 1.4 mg of oil, and left under magnetic stirring (500 rpm) during 30 min at 25° C. Then, 1.8 g of dispersion (copolymer, drug and oil) was added dropwise with a Pasteur pipette to 40 g of milli-Q water and left under stirring (500 rpm) during 2 h, at 25 °C. Finally, the dispersion was rotoevaporated under reduced pressure at temperature of 39 ± 1 °C, until a final mass of 4 g.

HPLC-UV Results

A typical HPLC chromatogram of TMC is shown in the insert of Figure S2, with a retention time of 6.18 min. As can be seen, a good linearity was obtained for the calibration standard curve of TMC in the concentration range between 0.5 and 10 µg mL-1. A linear relationship was established for the injected concentration ranges versus the peak area (y=3134+41685x) and R^2 was higher than 0.9988. The method's selectivity was confirmed by the absence of interferences at the retention time of TMC in HPLC chromatogram obtained for nanoparticle without drug (blank NP-11) (see Figure S3). Finally, regarding the method's sensitivity low values for both limit of detection (LOD = 0.041 µg mL-1) and limit of quantification (LOQ = 0.123 µg mL-1) were obtained. Therefore, the outcomes confirm that the proposed HPLC-UV method is suitable and was then used for determination of the encapsulation efficiency, drug loading and release kinetic profile.

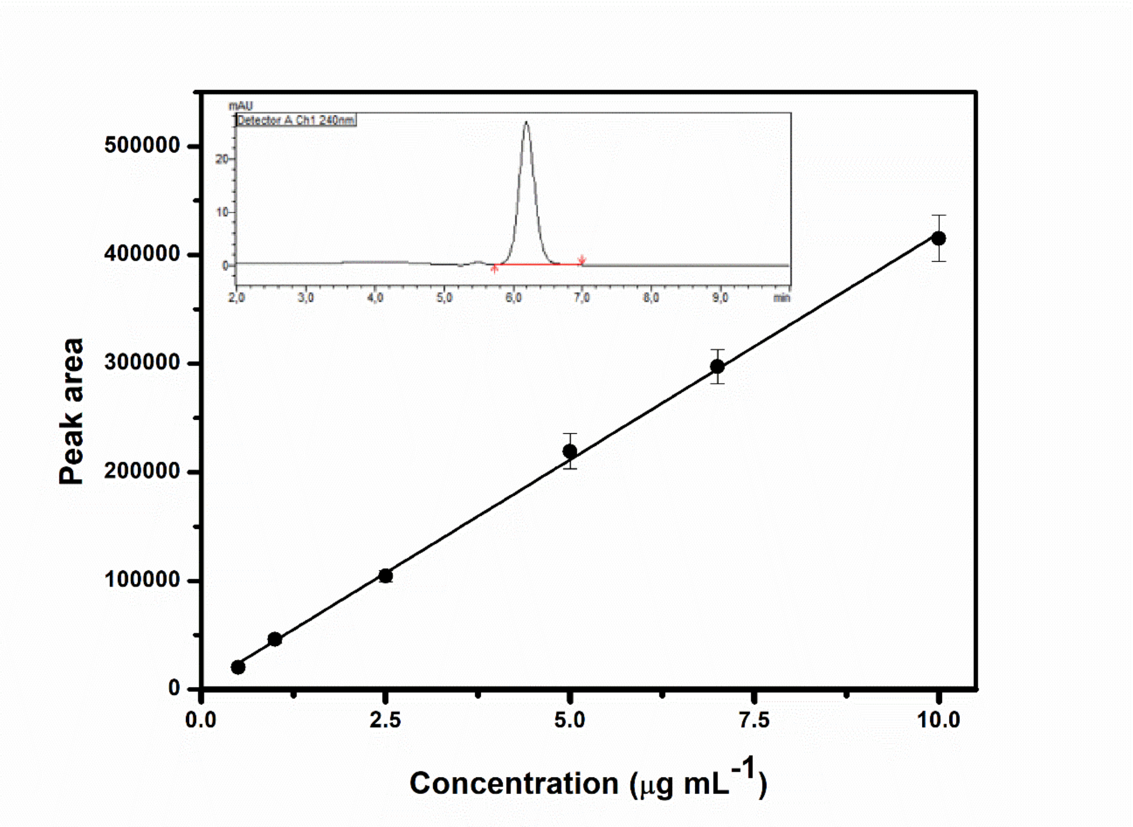

Figure S2. Calibration standard curve for TMC. Insert: HPLC chromatogram of TMC.

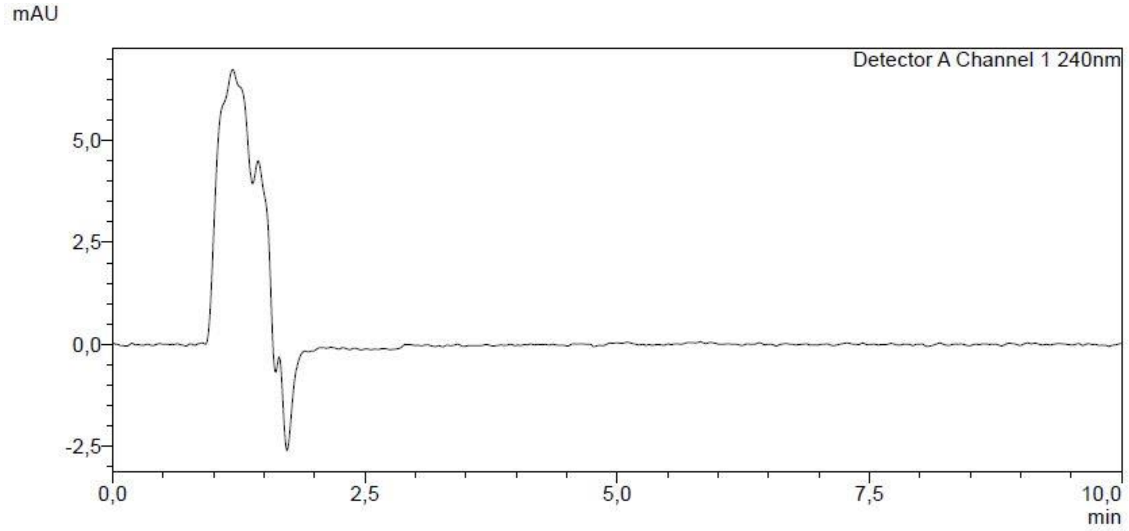

Figure S3. HPLC chromatogram for nanoparticle without drug (blank NP-11).
